# Natural Bioactive Compounds As Protectors Of Mitochondrial Dysfunction In Cardiovascular Diseases And Aging

**DOI:** 10.3390/molecules24234259

**Published:** 2019-11-22

**Authors:** Diego Arauna, María Furrianca, Yolanda Espinosa-Parrilla, Eduardo Fuentes, Marcelo Alarcón, Iván Palomo

**Affiliations:** 1 Thrombosis Research Center, Medical Technology School, Department of Clinical Biochemistry and Immunohaematology, Faculty of Health Sciences, Interdisciplinary Center on Aging, Universidad de Talca, Talca 3460000, Chile; araunadiego@gmail.com (D.A.); malarcon@utalca.cl (M.A.); 2 Thematic Task Force on Aging, CUECH Research Network, Santiago 8320000, Chile; maria.furrianca@umag.cl (M.F.); yolanda.espinosa@umag.cl (Y.E.-P.); 3 Departamento de enfermería, Universidad de Magallanes, Punta Arenas 6200000, Chile; 4 Laboratory of Molecular Medicine —LMM, Center for Education, Healthcare and Investigation—CADI, Universidad de Magallanes, Punta Arenas 6200000, Chile; 5 School of Medicine, Universidad de Magallanes, Punta Arenas 6200000, Chile

**Keywords:** cardiovascular diseases, aging, natural compounds, mitochondria

## Abstract

Diet, particularly the Mediterranean diet, has been considered as a protective factor against the development of cardiovascular diseases, the main cause of death in the world. Aging is one of the major risk factors for cardiovascular diseases, which have an oxidative pathophysiological component, being the mitochondria one of the key organelles in the regulation of oxidative stress. Certain natural bioactive compounds have the ability to regulate oxidative phosphorylation, the production of reactive oxygen species and the expression of mitochondrial proteins; but their efficacy within the mitochondrial physiopathology of cardiovascular diseases has not been clarified yet. The following review has the purpose of evaluating several natural compounds with evidence of mitochondrial effect in cardiovascular disease models, ascertaining the main cellular mechanisms and their potential use as functional foods for prevention of cardiovascular disease and healthy aging.

## 1. Introduction

The incidence of cardiovascular diseases (CVD) (i.e., acute myocardial infarction, cerebrovascular disease, and peripheral arterial thrombosis] has significantly increased in recent years. According to the World Health Organization, CVD will be the cause of 23.3 million deaths for the year 2030 [1,2]. The development of CVD is triggered by the presence of classic cardiovascular risk factors (smoking, dyslipidemia, hypertension, diabetes and overweight/obesity) [3,4]; however, these can be avoided by incorporating protective factors. In this sense, the Mediterranean diet one of the main protective factors against the development of CVD [5]. Epidemiological clinical trials have shown that changes towards a healthy diet should be a key factor in the prevention of CVD [6] and therefore a return in disease-free lifespan. 

The basis of CVD involves damage and remodeling of blood vessels that can result in blood flow restriction affecting the heart, brain and different organs [6]. In the majority of these cases, the development and progression of CVD are characterized by the interaction of atherosclerotic lesion and thrombus formation processes [7,8]. During these processes, the activated platelets and endothelial cells generate a variety of intracellular reactive species of oxygen [ROS] [9,10], that being overproduced can induce intracellular damage; producing phenomena such as mitochondrial dysfunction [11]. The mitochondria are crucial in processes such as oxidative phosphorylation and energy production but they are also essential for regulating other biological functions such as calcium homeostasis, oxidative response, and apoptosis [12]. All these processes are key in aging and are physiopathological components and therapeutic targets of cardiovascular diseases so that mitochondrial dysfunction can enhance both the onset of cardiovascular diseases and their clinical complications [11]. Different families of natural compounds have been proposed as potential protectors of mitochondrial function or as factors that lead to "recovering"; being some tested in animal models of CVD and in clinical trials [13,14]. Although mitochondrial antioxidants such as mitoquinone (MitoQ) have had promising results in the vascular area [15], effectiveness of this and other natural mitochondrial protectors is still unknown. We have set out to collect and evaluate the evidence for several natural compounds belonging to the families of anthocyanins, quinones, isothiocyanates and senolytic agents; which have been reported as protective of mitochondrial function, giving emphasis to their possible uses in the therapy (or prevention) of CVD and healthy aging. 

## 2. Oxidative Stress in Cardiovascular Diseases

One of the main physiopathological components of CVD is oxidative stress; being a treatment target for cardiac and vascular diseases [12]. ROS are produced by all vascular layers, including endothelium, smooth muscle, and adventitia [16]. Under physiological conditions, ROS act as signaling molecules that regulate vascular smooth muscle cell contraction, relaxation, and growth [17]. Pathophysiological conditions, such as the participation of cardiovascular risk factors [17,18,19], induce an imbalance between oxidants (ROS) and antioxidant defenses; causing an increase of the circulating and intracellular oxidative stress [20]. This leads to several alterations in endothelial cells at the level of proteins, lipids, mitochondria, and DNA [21]. The endothelium has an essential role in the regulation of vascular tone, promotion or inhibition of vascular growth, platelet aggregation and coagulation [22,23]. Within these processes, the production of intracellular and extracellular ROS play key roles [24]. The endothelial dysfunction (ED) is a pathological state of the vascular endothelium, which was identified such as a robust predictor of CVD. In this alteration, ROS is considered as signaling molecules that contribute to ED [25].

The common development of CVD is initiated by ED, followed by an atherosclerotic lesion; which may end up in a thrombotic process and an ischemic tissue injury [26,27]. Also, at this level, the activated platelets and endothelial cells generate a variety of intracellular ROS including superoxide anions, hydrogen peroxide, hydroxyl radicals, and peroxynitrite (ONOO-), which induce high oxidative stress damage [28,29,30]. At cardiovascular level, we can identify different pathways that produce and release ROS. The enzymatic sources of ROS well described in CVD are NAD[P]H oxidase (NOX) [31], cyclooxygenase (COX) [32], xanthine oxidase (XO) [33], uncoupled nitric oxide synthases (u-NOS) [34], and mitochondrial respiration; being the latter a new source for therapeutic objectives [35,36,37]. 

NOX is a multicomplex protein (seven members) that is present in the cellular membrane and mitochondria, being another important source of ROS production [38]. The activation of NOX results in the generation of superoxide anion and hydroxide peroxyde [39].

In relation to CVD, NOX-1 is upregulated in processes such as diabetic atherosclerosis [40], hypertension and restenosis [41]. Likewise, NOX-1 deletion in ApoE-KO mice reduces atherosclerosis. Similarly, the NOX-2 deletion has also been shown to be related to a reduction in atherosclerosis in descending aorta in mouse models [42], and the selective inactivation of NOX-2 causes regression of vascularization and the size and stability of atherosclerotic plaques [43]. The role of NOX-4 in atherogenesis is controversial, reporting that its inhibition or knockdown increases the formation of the atherosclerotic plaque [44,45]. NOX-5 was found to be upregulated in diabetes, hypertension, and human atherosclerotic lesions; however, recently it is described that NOX-5 has an important role in angiotensin II-induced vascular dysfunction and remodeling, but not in the development of hypertension [46,47].

Cyclooxygenase (COX) (two isoforms, COX-1, and COX-2) catalyzes the conversion of arachidonic acid [AA] into prostaglandin H2 [PGH2], which is subsequently converted by synthases and isomerases to the prostanoids PGE2, PGI2, PGF2a, and thromboxane A2 (TXA2) [48]. The role of AA metabolites generated by COX in homeostasis of vascular tone and in the clotting cascade has been well described [49]. The main objective of COX isoforms is the regulation of the balance between vasoconstriction and vasodilation, important processes for prothrombotic events [50]. Thromboxane A_2_ (TXA2) is a platelet-derived and is the predominant arachidonic acid (AA) metabolite in platelets, being an important physiological agonist [51]. 

Xanthine oxidases are found in endothelial cells and plasma and generate superoxide anions and hydrogen peroxide [52]. Their expression are increased in human atherosclerotic plaques, and their inhibition produces an attenuate effect in atherosclerotic plaques in mice, and a delay the progression of renal dysfunction in adult hypertensive patients with hyperuricemia [53,54,55]. 

NOS is an enzyme that catalyzes oxide nitric (NO) production. NO plays a vasoprotective role and is constitutively expressed in endothelium by activation of eNOS [56]. Under conditions of oxidative stress, eNOS becomes dysfunctional [uncoupled form] [57]. Superoxide anions generated by other enzymes combine with NO to form a highly reactive ROS called peroxynitrite (ONOO-) [58]. Uncoupled eNOS produces superoxide anions instead of NO and hence is a ROS generator [57]. Studies have shown an inverse relationship between the availability of cofactors of eNOS and development of endothelial dysfunction [59,60]. Besides the contribution in oxidative stress of the different enzymatic systems described above, mitochondria are key organelles in the regulation of oxidative stress and therefore they are regarded as novel therapeutic targets for CVD [11]. 

## 3. Mitochondrial Dysfunction in CVD

Mitochondria are semi-autonomous organelles composed by a double membrane, matrix internal and internal DNA. The size generally varies from 0.75 to 8 μm in cardiomiocytes [61]. An appropriate mitochondrial functioning is necessary for the correct performance of tissues and organs with high energy demand, such as the heart and brain [62]. Mitochondrial dysfunction is found in the axis of ROS overproduction in CVD. It is well know that the deregulation in the production of ROS, produces a decrease of the mitochondrial membrane potential, and this leads to activation of a number of signaling proteins, inducing apoptosis [11]. Mitochondria is very sensitive to alterations in the nutrients and oxygen supply, adapting their metabolic activity according to changes in the intra and extracellular environment [63]. In CVD, this adaptation is damaged, which leads to a progressive decline of the mitochondrial function associated with abnormalities in the respiratory chain and ATP synthesis, increased oxidative stress, and loss of the structural integrity [64]. 

In addition, CVD is linked to a giant mitochondria formation [65]. These giant mitochondria are generated for overexpression of fusion proteins (Mnf1-2), being usually dysfunctional and eliminated by mitophagy process. Mitophagy mechanism is altered CVD, maintaining the abnormal functioning and extending cell damage [36]. Likewise, an increase in mitochondria fragmentation due to the positive regulation of dynamin protein 1 (Dnp1) has been observed in ischemia-reperfusion model [66]. This protein can form pro-apoptotic complexes with the Mnf2 and Bax proteins in the mitochondrial outer membrane, inducing the formation of mitochondrial transition pores (mPTP) and the release of cytochrome C [67]. 

Mitochondrial dysfunction is a key part of the atherogenic molecular mechanism, with mitochondrial functioning being crucial for the production of nitric oxide, calcium management and apoptosis for vascular endothelial cells. It has been suggested that NOX-4, but not NOX-1/2, and mitochondrial oxidative stress are mediators of CVD in aging, therefore regulating NOX-4 activity/expression and using mitochondrial antioxidants are potential approaches to reduce aging-associated CVD [68]. 

It has been observed that low-density lipoprotein (oxLDL) induces a decrease in mitochondrial activity producing ATP, stimulating hyperplasia, migration, and proliferation of VSMC; promoting the formation of atherogenic plaque. On the other hand, it has been described that the hyperplasia suppressor gene (HSG), a murine protein homologous to the human protein mitofusin 2 (Mfn 2), blocks the proliferation of VSMC. Mfn 2 is a key protein in the process of mitochondrial fusion and Ca^2+^ signaling [69], could be important for targeting dysfunctional mitochondria to mitophagy [70].

Likewise, it is well known that hypoxia present in cardiac ischemia induces mitochondrial dysfunction, having an important role in the molecular mechanism of ischemia-reperfusion injury [71]. This injury induces an increase in intramitochondrial calcium, activating ROS production mechanism, blockade of the electron transport chain and loss of mitochondrial membrane potential [72]. In models of ischemia-reperfusion, it has been observed that the activation of mitophagy is an adaptive response in cardiac damage, for which the regulation of this mitochondrial mechanism could improve the prognosis of patients with CVD [73,74,75].

In cardiac hypertrophy, the mitochondrial fission has a preponderant role in the progression of the disease [76,77]. In cases of heart failure, a different context is observed, where the mitochondria are damaged due to membrane rupture and exposition of their matrix [78]. This damage greatly affects the capacity limit of ATP synthesis and activates mechanisms that induce oxidative stress and increase calcium flow; inducing the phenomenon of dysfunction [64]. A decrease in the activity of complexes I and IV, as well as key enzymes of the Krebs cycle, have been observed in subjects with heart failure [79]. In addition, in the cardiomyocytes of these patients, it has been observed that the replicative capacity of mitochondrial DNA is affected, which decreases the expression of proteins in response to oxidative stress [79].

## 4. Mitochondrial Dysfunction in Aging: Crossing Point with CVD?

Aging is a major risk factor for the occurrence of acute cerebrovascular accidents and CVD, such as stroke and myocardial infarction [80]. In addition, the aging process associated with a chronic state of high oxidative stress-mediated by complex and interconnected pathways, being the mitochondria a cross point [64,81]. The aging process produces alterations in the cardiovascular system as cardiac hypertrophy, alterations in ventricular capacities, increased arterial stiffness, and impaired endothelial function [82]. In all these alterations, oxidative stress plays a central role, because cardiomyocytes and endothelial cells lose part of their antioxidant response capacity and increase ROS production [83]. The overproduction of ROS would result from an alteration of mitochondrial function and transcriptional activity, resulting in an increase of mutations in mitochondrial DNA. This DNA damage produces extense cellular damage and a reduction in life expectancy [84]. Among the most accepted measures for healthy aging, is the caloric restriction, which is known to have a protective effect in the cardiovascular system mediated by mitochondrial function [83]. 

The aging process is characterized by seven general hallmarks: Genomic instability, telomere wear, epigenetic alterations, loss of proteostasis, detection of deregulated nutrients, mitochondrial dysfunction, cellular senescence, exhaustion of altered stem cells and intercellular communication [85]. Mitochondrial dysfunction has been evidenced in aging by an increase of mutations on mitochondrial DNA, and a decrease in activity of mitochondrial enzymes, respiratory capacity per mitochondria and phosphocreatine recovery time. In addition to those alterations, mitochondria present changes in morphology, biogenesis, and dynamics [86,87]. Alterations in mitochondrial dynamics, both in their fusion/fission processes, disrupt the regulation and coordination between the generation and removal of damaged mitochondria, which propagates the formation of dysfunctional mitochondria [36,86]. The damaged or dysfunctional mitochondria are selectively degraded by a mitochondria-specific autophagy process called mitophagy, whereas new mitochondria are synthesized by biogenesis process [88]. A correct interconnected system is necessary to maintain the balance between the mitophagy and mitochondrial biogenesis, being a key point to consider for healthy aging [64,89,90].

In consideration of the theory of free radicals, it would be suggested that the increase of intramitochondrial oxidative stress [due to overproduction of ROS] would be the initial mechanism for transcriptional deregulation and the increase of mutations in mitochondrial DNA [87,91]. However, there is still not enough robust evidence to establish a causal relationship between mitochondrial dysfunction and aging [87]. This is because most of the data on mitochondrial functioning in aging have been developed in murine models, which do not present a good correlation and replication with what was observed in normal human aging [92].

Likewise, it has been described that mitochondrial dysfunction participates in numerous age-related pathologies including neurodegenerative and cardiovascular disorders, diabetes, obesity, and cancer; which makes it difficult to elucidate if mitochondrial dysfunction would be the cause or a consequence of aging [93,94,95]. In relation to CVD, mitochondrial dysfunction plays a key role both for the development of the disease and new therapeutic approaches [64,73,75]. 

## 5. Bioactive Compounds with Mitochondrial Protective Function

**Anthocyanins.** There exists a growing interest in the therapeutic effects of polyphenolic compounds known as anthocyanins, which are water-soluble pigments, found in colored berries, fruits, and vegetables [96]. These secondary metabolites are known for their antioxidant properties and currently, they have been associated with a lower risk of CVD [97]. The mechanisms that explain these possible benefits are heterogeneous. For example, a reduction of mitochondrial stress has been indicated as one of the possible targets of these compounds [98], this would be due to the critical role of mitochondria in the regulation of homeostasis in heart [99] and endothelium [100]. 

One of the main lesions that occur during ischemia/cardiac reperfusion is the inhibition of complex I of the electron transport system, which in turn causes the suppression of ATP production in the oxidative phosphorylation process [101]. However, delphinidin [100] and cyanidin anthocyanins [102] can mitigate these effects, because they are able to increase the activity of complex I, since both can serve as electron acceptors (instead of CoQ 1) in the oxidation of NADH, restoring at least partially the inhibition induced by ischemia in complex I [103]. These findings support the idea that anthocyanins possessing two or three hydroxyl groups show a beneficial effect on oxidative phosphorylation; in contrast, anthocyanins with only one hydroxyl group have little effect [104]. Similarly, recent research reports that anthocyanins: cyanidin 3-O-glucoside and delphinidin 3-O-glucoside can protect heart against injury induced by ischemia/reperfusion by activating signal transduction pathways and maintaining mitochondrial functions instead of acting only as antioxidants [105]. This is based on the location of STAT proteins, since they are not only found in the nucleus and cytoplasm of cardiomyocytes, but they are also described in the mitochondria [106]. Another event that causes mitochondrial damage from cardiac ischemia or reperfusion, is the loss of cytochrome C (cyt c) by mitochondria, process that induces apoptosis of cardiomyocytes due to the fact that cyt c causes the activation of caspases 9 [107]. However, this activation is regulated by the redox state of cyt c, since its oxidized state is more potent in the activation of caspases than its reduced state [108]. Given the above, anthocyanins are described as capable of blocking the ischemia-induced apoptosis in the perfused heart and potentially favor mitochondrial respiration by reducing cytosolic cyt c. Specifically, cyanidin prevents the activation of caspases induced by ischemia [109,110], as demonstrated in a test conducted in rat heart, where cyanidin shows a protective effect related to its high capacity to reduce cytosolic cyt c [110]. 

Classic cardiovascular risk factors cause oxidative stress which alters the capacity of endothelial cells and leads to so-called endothelial “dysfunction” that reduces their ability to maintain homeostasis and leads to the development of pathological inflammatory processes and vascular diseases such as atherosclerosis [111]. It is described that the delphinidin anthocyanin protects against mitochondrial dysfunction induced by oxLDL in vascular endothelial cells, by suppressing the opening of the mitochondrial permeability transition pore and the loss of potential mitochondrial membrane [ΔΨm]. This causes the decrease of ROS and the generation of superoxide anions, which blocks the mitochondrial apoptotic pathway and prevents endothelial dysfunction because the endothelial cells are able to absorb the delphinidin via the transporter SGLT1 [112].

In the same way, delphinidin can attenuate the mitochondrial damage produced by the oxLDL in atherosclerotic lesions by reactivating mitochondrial enzymatic activities in porcine aortic endothelial cells. The oxLDL, reduces the activity of the mitochondrial respiratory chain complex [I-IV] and decreases the content of NADH dehydrogenase (ND) 1, ND6 (subunits of the complex I enzyme) or cytochrome b (subunit of the complex III enzyme) [113]. In addition, it is described that cyanidin normalizes the inhibition that glycosylated low-density lipoprotein produces (glLDL), on the enzymatic activities of the mitochondrial electron transport chain in porcine endothelial cells. As a result, the activities in Complex I and III become normal, as well as the abundances of NADH dehydrogenase 1 in Complex I and cytochrome b in Complex III in vascular endothelial cells [102]. 

Although there is a vast data of studies that support the benefits of the use of anthocyanins in ischemia-reperfusion models, the mechanism of mitochondrial antioxidant action or transcriptional regulation is still unclear [105]. Also, has been described that anthocyanins would reach a low bioavailability and serum concentration, being a problem for therapeutics considerations [114]. Despite these disadvantages, anthocyanins are the most abundant flavonoids in fruits such as berries; one of the functional foods in vogue today [105]. The most common and studied anthocyanins that provide mitochondrial protection activity are shown in Table 1. Recently, a review addressed the effects of anthocyanin consumption on cardiovascular risk factors, considering retrospectives, prospectives and cross-sectional studies [115]. Briefly, the data provide for this study, shows a solid evidences that higher intakes of anthocyanins improve arterial stiffness [assessed by pulse wave velocity] and blood pressure, however, ignorance of the role of the intestinal microbiome in the metabolism and absorption of anthocyanins produces a high variation of the effects of these molecules on cardiovascular health [115,116]. On the other hand, studies in humans have shown a direct relationship between a high consumption of anthocyanin and a decrease in the risk of CVD development, observing a 32% decrease in the risk of myocardial infarction and a decrease in CVD mortality [117,118,119].

**Quinones.** Quinones are secondary metabolites that occur predominantly in angiosperms, fungi, lichens, and bacteria that perform a variety of functions in plants, intervening in oxidative stress, pathogen protection, and redox systems [122]. These natural metabolites have been shown to exhibit potent anticancer, anti-inflammatory, antimicrobial, antioxidant and cardioprotective properties [123,124,125]. 

In view of the relevance of quinones in the treatment of CVD, diverse mechanisms of action on the use of these compounds have been proposed [126,127,128]. As an example, it is described that the pyrroloquinoline [quinone redox cofactor for bacterial dehydrogenases], preserves mitochondrial function and prevents oxidative injury in rat cardiac myocytes, through its action as a free radical scavenger [126] and being highly effective in reducing infarct size and improving hemodynamics in rats subjected to I/R injury through reducing the death of the myocardiocytes [124,129]. 

Thymoquinone is a quinone that is the most abundant constituent of the volatile oil of *Nigella sativa* seeds but it is also found in *Eupatorium ayapana*, *Calocedrus decurrens*, *Thymus vulgaris*, *Nepeta distans* and *Origanum* and *Satureja* species [130]. This compound attenuates cardiotoxicity in rats through a mechanism related to its ability to decrease oxidative stress and improve mitochondrial function through an increase of ATP production [131]. The activation of the mitochondrial mechanisms may be due to stimulation of *Nrf2*, which regulates mitochondrial function and promotes the expression of antioxidant enzymes to reduce oxidative damage [132]. In addition, it preserves the activity of various antioxidant enzymes such as catalase, glutathione peroxidase, and glutathione-S-transferase [133].

Ubiquinone, a natural constituent in tobacco plant [*Nicotiana tabacum*] that is mainly distributed in the membrane of the mitochondria [134], is vital for the functioning several organs including the heart [135]. It is known that cardiac failure is related to decreased energy production by the mitochondria [136]. Ubiquinones, participate in the process of oxidative phosphorylation and maintain cellular energy in the cardiac muscle under conditions of metabolic stress causing a decrease in myocardial damage [137]. The protection generated by this compound can be due to its participation in the transport of electrons from organic substrates to oxygen in the respiratory chain of mitochondria with the production of energy, performing a role in providing energy for the functioning of the failing and energy depleted heart [138]. Experimental investigations report that the ubiquinone in rats improvement of myocardial energy expenditure and oxidative stress induced by carbon tetrachloride [139]. The reviews that addressed the efficacy of ubiquinone supplementation for the prevention of CVD, show a significant reduction in systolic blood pressure without improvements in other CVD risk factors; while that in patients under cardiac surgery demonstrates a decrease in the need for inotropic drugs and risk of ventricular arrhythmias [140,141]. However, exists a discrepancy in the studies that evaluate the effects of ubiquinone supplementation in the short and long term, with null effects in the improvement of ventricular ejection fraction in heart failure but with positive effects in the reduction of risk and number of cardiovascular events [142,143,144].

In general, the beneficial effects of these phytochemical compounds are attributed to their antioxidant properties as well as their ability to improve the mitochondrial function and energy production in heart tissues. The most common and studied quinones that provide mitochondrial protection activity are shown in Table 2.

**Isothiocyanates.** Isothiocyanates are natural highly reactive organosulphur synthons [145] present in cruciferous vegetables such as broccoli, rocket, cauliflower, Brussels sprouts, cabbage, radish, turnip and watercress being responsible for the plant sharp taste that is linked to their defense system [146]. Recent reviews have reported therapeutic and prophylactic properties for these compounds, having antioxidant, anti-inflammatory, antimicrobial, neuroprotective and cardioprotective activities [147,148]. Among their cardioprotective functions, different mechanisms of intracellular action have been detailed, being a common point the strengthening of the antioxidant response [149].

The main compounds reported in the literature with cardioprotective function are: Sulforaphane, Glucoraphanin, Glucomoringin-isothiocyanate, and Sinigrin. This group of molecules is biologically produced by hydrolysis of the precursor Glucoraphanin by the action of myrosinases [β-thioglucosidases] at the time of chewing or cooking the container food [146]. Sulforaphane is obtained from cruciferous vegetables such as broccoli, brussels sprouts, and cabbages. In a model of murine reperfusion ischemia, it has been reported that the addition of Sulforaphane in the diet [5mg / kg for 3 days] produces an enhanced expression of Nrf2, HO-1, and NQO-1, attenuating the expression of inflammatory and apoptotic markers [146].The increase in the expression of Nrf2 produces a gene regulatory response resulting in the expression of antioxidant enzymes and glutathione biosynthesis, being a fundamental axis against oxidative stress [150]. In another study that used a murine model of photo-induction of thrombosis, it was shown that the addition of sulforaphane to the diet [5 or 50 mg/kg, 24 hours prior to induction of thrombosis] decreased the formation of thrombi in the cerebral microcirculation [151]. Glucomoringin-isothiocyanate [GMG-ITC] is an uncommon member of glucosinolate group belonging to the Moringaceae family, of which *Moringa oleifera Lam*. is the most widely distributed. In a murine model of cerebral ischemia/reperfusion, the addition of GMG-ITC [3.5 mg, 24 hours prior to induction of ischemia], was shown to exert neuroprotective properties in preventing ischemia-induced damage and the related cascade of inflammatory and oxidative mediators that exacerbate the progression of this disease in an experimental rat model [4]. Sinigrin is a glucosinolate that belongs to the family of glucosides found in some plants of the Brassicaceae family such as Brussels sprouts, broccoli, and the seeds of black mustard. It has been reported that the treatment with sinigrin [10 mg/kg, three times a week for 16 cycles] prevented atherosclerosis in ApoE^−/−^ mice. The preventing effect of sinigrin may be due to the ability to suppress the production of inflammatory cytokines and the expression of pro-atherogenic factors in serum, aorta, and liver tissue [inhibits the VCAM-1 expression via preventing NF-κB and p38 MAPK/JNK pathways in MOVAS cells] [7]. Clinical trials that have used isothiocyanate supplementation by increasing food consumption with high concentration of these compounds, shown a decrease in LDL levels and an improvement in blood pressure control [152,153]. Recently, in a rabbit model of cardiac failure, the supplementation with Sulforaphane [0.5 mg/kg in a subcutaneous dose for five days per twelve-week] improved ventricular cardiac function and remodeling by inhibiting oxidative stress and inflammation with a possible mitochondrial role [154]. Table 3 shows in detail the main findings regarding isothiocyanates that provide mitochondrial protection activity.

**Senolytic agents.** Within the different hallmarks of aging, there is cellular senescence [158]. This phenomenon lies in the arrest of cell division and the accumulation of arrested cells in different tissues, which finally produces a propagation of cell damage and loss of hemostasis [158]. Currently, the phenomenon of senescence has been described as a physiopathological component of CVD, observing an increase of these cells at the endothelial, cardiac and atherosclerotic plaques in patients with CVD [159]. Also, cellular senescence entities have been associated with mitochondrial dysfunction, which is another hallmark of aging and a pathological factor of CVD [64,160]. Currently, there is a vast literature that argues that the elimination of senescent cells could slow the progression of cell damage associated with aging and prevent or treat CVD [161,162,163,164]. Accumulation of senescent cells, both at the vascular or/and cardiac level, has been associated with an increased risk of atherosclerosis, heart failure, dilated cardiomyopathy, and myocardial fibrosis. [161]. Faced with this challenge, the senolytic agents have appeared as an alternative for the elimination of these senescent cells [165]. A senolytic agent is defined as a small molecule capable of selectively removing senescent cells [166]. Regarding the natural senolytic compounds, the most studied having beneficial cardiovascular effects are: Quercetin, fisetin and piperlongumine [166]. 

Quercetin is a flavonoid widely found in vegetables and fruits, as well as in beverages such as tea and red wine [167]. In vitro studies showed different effects of quercetin as anti-inflammatory, antioxidant, anticlotting, and vasodilatory properties; which have been described as beneficial for the prevention and treatment of CVD [168,169,170]. The mechanisms by which it exerts these effects are heterogeneous, however, the regulation of mitochondrial functionality appears as one of the main protective mechanisms [171]. Quercetin inhibits the platelet aggregation in humans and prevents the endothelial dysfunction in vitro and in apolipoprotein E knockout mice [172,173,174]. Both mechanisms are intrinsically related to the development of CVD [26]. The ingest of quercetin [150 and 300 mg] exerts an antiaggregant effect against collagen induction by a mechanism dependent of partial inhibition of tyrosine phosphorylation of the tyrosine kinase Syk and phospholipase Cγ2, components of the platelet glycoprotein VI collagen receptor signaling pathway [172]. The endothelial protective effects of quercetin have been described to be mediated by different mechanisms, among them, the increase of the bioavailability of nitric oxide [in vitro model], inhibition of superoxide production and regulation of expression of p47phox [key protein for NOX activation] [173]. One of the main beneficial effects of quercetin is related to the regulation of blood pressure and induction of vasorelaxation, in which a mechanism dependent on inhibiting the overexpression of p47phox and the subsequent increased O2− production, resulting in increased nitric oxide bioavailability [173]. This positive effect of quercetin was also observed in hypertensive humans, however, with no effect on the level of circulating oxidative stress; which is contradictory with what was observed in animal models and in vitro [175]. Finally this protective effect was checked in a clinical trial doubled blinded that used dose quercetin at 150 mg/day for 6 weeks [168]. This study showed that overweight subjects with a high-cardiovascular disease risk phenotype present a reduction in systolic blood pressure and plasma oxidized LDL levels [168]. In this same sense, in an in vitro model of posttraumatic cardiac dysfunction in H9c2 cells, quercetin may reverse by reducing cardiomyocyte apoptosis through the suppression of TNF-α increases, ROS overproduction and Ca2+ overload in cardiomyocytes, representing a potential preventive approach for the treatment of secondary cardiac injury after mechanical trauma [176]. The cardioprotective effects of quercetin are achieved by reducing the activity of Src kinase, signal transducer and activator of transcription 3 [STAT3], caspase 9, Bax, intracellular ROS production, and inflammatory factor and inducible MnSOD expression [177]. Both STAT3, as well as the release of caspases and Bax, are controlled at the mitochondrial level. On the other hand, the production of ROS in cardiomyocytes is mainly contributed by the mitochondria and NOX proteins, for which quercetin could exert mitochondrial protection by regulating these intramitochondrial processes [12,64].

Fisetin is another flavonoid which exists in various fruits and vegetables such as onions, apples, grapes, persimmons, strawberries, and cucumbers [178]. This compound has not reported any toxicity in high doses, its consumption being safe in humans [179]. It well described that fisetin exerts a series of beneficial biological activities, including antitumor, antioxidant, anti-inflammatory, antiangiogenic, hypolipidemic and neuroprotective effects [180,181]. The high senolytic activity of fisetin has been the most striking feature for its potential use in CVD and against aging [182]. Fisetin has presented senolytic and anti-inflammatory activity in HUVECS endothelial cells, inducing apoptosis in senescent cells and without affecting cell proliferation [182,183]. Both the decrease of senescent endothelial cells and the maintenance of their proliferation have been described as protective mechanisms against CVD [162]. On the other hand, it has been described that fisetin would activate the transcription activity of Nfr2 in HUVECS, exerting an antioxidant response focused on the activity of heme oxygenase-1 [184]. It is important to note that recently it has been reported that fisetin would confer cardioprotection in vitro model of myocardial ischemia-reperfusion injury [Langendorff isolated heart perfusion system] by suppressing mitochondrial oxidative stress and mitochondrial dysfunction mediated via inhibition of glycogen synthase kinase 3β [185]. The inhibition of this pathway provides cardioprotection by modulating mitochondrial ATP-sensitive K+ channel, the mammalian target of rapamycin [mTOR] signaling pathway, and autophagy [186]. This shows that fisetin would exercise both a senolytic activity and mitochondrial protection; mechanisms for which one could also justify its beneficial effects on aging [90,164].

Piperlongumine is an alkaloid found in the long pepper species [*Piper longum L*.] [187]. This compound presents a well-documented antiplatelet aggregation, anti-inflammatory, and anticancer effects [166]. Both its aggregation inhibiting activity and the reduction of inflammation are beneficial for the prevention of CVD and against aging [181,182]. Iwashita et al, in year 2007, describe that Piperlongumine exerts a concentration-dependently inhibition activity of platelet aggregation induced by thromboxane A[2] receptor agonist U46619, in a rabbit model [EC50 value of 0.47 μM] [188]. Years later, in 2015, Yuan et al show that piperlongumine exert a dose-dependently inhibition of collagen-induced platelet aggregation, calcium influx, CD62p expression, microparticles formation and thrombus formation on collagen with maximal inhibition at 100 μM [189]. The mechanism inhibitory proposed of piperlongumine would be blocked the activation of JAK2 and STAT3 in collagen-stimulated platelets. STAT3 is a transcription factor, which does not possess nucleus platelets, exerts its transcriptional effect at the mitochondrial level [190]. The mitochondrial mechanism by which STAT3 would exert this inhibition of aggregation is still unknown since this effect would not be mediated by ROS [189]. In a study by Dong Ju Son et al, they set out to evaluate the anti-atherosclerotic potential of this compound in an in vivo murine model of accelerated atherosclerosis and defined its mechanism of action in aortic VSMC in vitro [191]. In this murine model of induced atherosclerotic lesion, piperlongumine significantly reduced atherosclerotic plaque formation, proliferation of endothelial cells and induce nuclear factor-kappa B [NF-κB] activation in these cells [191]. However, this antiatherosclerotic effect has not been observed in humans and conclusive information about its tolerance is lacking [192]. Piperlongumine treatment in VSMC showed inhibition of migration and platelet-derived growth factor BB [PDGF-BB]-induced proliferation [191]. PDGF-BB exercises regulation of mitochondrial fission in VSMC in vitro, so piperlongumine would play a role of mitochondrial protection in hyperproliferation vascular phenomena [193]. Table 4 shows in detail the main findings regarding senolytic agents that provide mitochondrial protection activity are shown in Table 4.

## 6. Conclusions

A broad record of evidence based on the study of cardiovascular disease models agrees on a therapeutic effect for several natural bioactive compounds against CVD. Among them, anthocyanins, quinones, isothiocyanites and senolytics are the most visible and probably the most promising compounds showing an antiaging effect. Even though the mechanisms that explain the possible benefits of these molecules are heterogenous, they are mostly explained by their ability to either regulate oxidative phosphorylation, the production of ROS, the expression of mitochondrial proteins or selectively remove senescent cells. All together their use is supportive as functional foods against cardiovascular disease and as promoters of healthy aging.

## Figures and Tables

**Table 1:** The most common and studied anthocyanins compounds that provide mitochondrial protection.

Pathology/Model	Compound	Effect	Reference
Hearth ischemia	Delphinidin-3-glucoside and Cyanidin-3-glucoside	Reduction of cytosolic cyt c directly and rapidly	[109]
Pre-perfusion of hearts	Cyanidin-3-glucoside	Prevention of ischemia-induced caspase activation	[109]
Pre-perfusion of hearts	Delphinidin-3-glucoside and Cyanidin-3-glucoside	Support of mitochondrial state 4 respiration even in the presence of exogenous cyt c	[109]
Ischemia and other diseases involving mitochondrial complex I dysfunction	Delfinidin-3-glucósido (Dp3G) y la cianidina-3-glucósido (Cy3G)	Action as electron acceptors in complex I-mediated oxidation of NADH	[103]
Endothelial dysfunction	Cyanidin- delphinidin- and pelargonidin-3-glucoside	Inhibition of several crucial signaling cascades, upstream and downstream of mitochondria.	[120]
Endothelial dysfunction	Malvidin-3-glucoside	NO balance and in inhibition of pro-inflammatory signaling pathways	[121]

**Table 2:** The most common and studied quinones compounds that provide mitochondrial protection.

Pathology/Model	Compound	Effect	Reference
Cardiac failure	Pyrroloquinoline quinone	Antioxidant activity in cardiac myocytes through its action as a free radical scavenger	[126]
Cardiac failure	Thymoquinone	Reduction of oxidative stress and improvement of mitochondrial function through increasing ATP production in cardiac myocytes	[131]
Cardiac failure	Ubiquinone	Increase of the transport of electrons from organic substrates to oxygen in the respiratory chain of mitochondria	[138]

**Table 3:** The most common and studied isothiocyanates that provide mitochondrial protection.

Pathology	Compound	Effect	Reference
Renal ischemia/reperfusion	Sulforaphane	Enhancement of the expression of Nrf2, HO-1, and NQO-1, attenuation of the expression of inflammatory and apoptotic markers	[150]
Photo-induced thrombosis model	Sulforaphane	Reduction of LPS-mediated enhancement of thrombus formation in the cerebral microcirculation	[151]
Cerebral ischemia/reperfusion	Glucomoringin-isothiocyanate	Reduction of TNF-alpha release, NFκBp65 nuclear translocation, markers of inflammation and oxidative stress	[155]
Murine AIDS model with heart dysfunction	Sulforaphane	Inhibition of apoptosis by increasing the Bcl-2/Bax ratio; Suppression of the expression of inducible nitric oxide synthase and inactivation of the cytoplasmic nuclear factor κB	[156]
Murine AIDS model with heart dysfunction	Benzyl Isothiocyanate	Inhibition of apoptosis by increasing the Bcl-2/Bax ratio	[156]
Murine AIDS model with heart dysfunction	Phenethylisothiocyante	Inhibition of apoptosis by increasing the Bcl-2/Bax ratio; Suppression of the expression of inducible nitric oxide synthase and inactivation of cytoplasmic nuclear factor κB	[156]
Atherogenic murine model ApoE knockout	Sinigrin	Reduction in serum concentrations of LDH, TC, LDL, and pro-inflammatory cytokines. Attenuated mRNA expression of adhesion molecules [VCAM-1 and others] and chemokines	[157]

**Table 4:** The most common and studied senolytic agents that provide mitochondrial protection.

Pathology/Model	Compound	Effect	Reference
Endothelial dysfunction model of thoracic aortae cell from male Wistar rats	Quercetin	Prevention of overexpression of the p47phox subunit of NOX. A decrease in O_2_^-^ production. Increase of bioavailability of NO	[173]
Posttraumatic cardiac dysfunction in H9c2 cells	Quercetin	Suppression of TNF-α increase of ROS overproduction and Ca2+ overload in cardiomyocytes	[176]
Ischemic/reperfusion model in H9C2 cells	Quercetin	Reduction of activity and activation of Src kinase, STAT3, caspase 9 and Bax. Decrease of intracellular ROS production, and expression of inducible MnSOD.	[177].
HUVECS endothelial cells in a hyperglycemic model	Fisetin	Induction of apoptosis in senescent cells without affecting cell proliferation.	[183]
HUVECS cells	Fisetin	Increase in the transcription activity of Nfr2, mainly HO-1 expression.	[184]
Myocardial ischemia-reperfusion injury [Langendorff isolated heart perfusion system]	Fisetin	Decrease of mitochondrial oxidative stress and mitochondrial dysfunction mediated by inhibition of glycogen synthase kinase 3β	[185]
Human platelets from healthy donors	Piperlongumine	Inhibition of collagen-induced platelet aggregation, calcium influx, CD62p expression, microparticles formation, and thrombus formation.	[189]
In vivo murine model of accelerated atherosclerosis	Piperlongumine	Reduction of atherosclerotic plaque formation, the proliferation of endothelial cells and induction of NF-κB activation, mediated by platelet-derived growth factor BB [PDGF-BB]- inhibiton.	[191].
